# ADHERENCE TO INTERVENTIONS AND COGNITIVE CHANGES IN FALLEN OLDER PEOPLE: RANDOMIZED CLINICAL TRIAL

**DOI:** 10.1093/geroni/igae098.2646

**Published:** 2024-12-31

**Authors:** Juliana Ansai, Graziele Norberto Pereira, Mariana Luiz de Melo, Mariana Ignácio Sossai, Leticia Teodoro Maciel, Mel Silva de sá, Ana Laura Oliveira Dias, Karina Gramani-Say

**Affiliations:** Federal University of São Carlos, São Carlos, Sao Paulo, Brazil; Federal University of São Carlos, São Carlos, Sao Paulo, Brazil; Federal University of São Carlos, São Carlos, Sao Paulo, Brazil; Federal University of São Carlos, São Carlos, Sao Paulo, Brazil; Federal University of São Carlos, São Carlos, Sao Paulo, Brazil; Federal University of São Carlos, São Carlos, Sao Paulo, Brazil; Federal University of São Carlos, São Carlos, Sao Paulo, Brazil; Federal University of São Carlos, São Carlos, Sao Paulo, Brazil

## Abstract

Falls are an important health challenge for older people and society. Understanding how to reduce cognitive risk factors for falls is essential to develop effective prevention strategies. The purpose of this work was to assess the impact of adherence to interventions on cognitive changes after 16 weeks in older people with a history of falls. A controlled, randomized, single-center clinical trial was conducted with brazilian community-dwelling older adults with a history of recurrent falls in the past 12 months. Participants were allocated to Intervention Group (IG) and Control Group (CG). The IG underwent case management, including multidimensional assessment, explanation of fall risk factors, proposal of intervention based on risks, development of an individual fall intervention plan, plan monitoring and review. The CG received monthly health-related guidance. The assessment included cognitive measures (Addenbrooke’s Cognitive Examination ACE-R, Mini-Mental State Examination, Verbal Fluency tests, Clock Drawing test CDT) at baseline and after 16 weeks, and adherence (frequency) to the intervention after 16 weeks. As results, the sample was composed by 55 participants (26 CG, 29 IG). The mean adherence to intervention was 80.27%. The IG improved the TDR performance, compared to the CG (p=0.034). Both groups presented a non-significant improvement in the ACE-R score over time. Adherence to the case management intervention did not influence changes in cognitive risk factors for falls (p>0.050). This findings point the need to understand better the factors related to improvement on risk factors for falls, to achieve personalized approaches in older people.

